# Mpox in a non-HIV immunosuppressed host: a case report from Nigeria

**DOI:** 10.11604/pamj.supp.2025.50.1.46082

**Published:** 2025-03-03

**Authors:** Chizaram Onyeaghala, Chioma Tochukwu-Onyejieme, Uche Tralagba, Bolaji Ibiesa Otike-Odibi, Omosivie Maduka, Datonye Alasia

**Affiliations:** 1 Department of Internal Medicine, University of Port Harcourt Teaching Hospital, Port Harcourt, Rivers State, Nigeria,; 2 Department of Community of Medicine, University of Port Harcourt Teaching Hospital, Port Harcourt, Rivers State, Nigeria

**Keywords:** Mpox, non-HIV immunosuppression, rheumatoid arthritis, Nigeria, case report

## Abstract

There is a lack of studies describing the impact of mpox in non-HIV immunosuppressed conditions such as severe autoimmune disorders, haematologic malignancies, solid organ transplant recipients, and those on immunosuppressive medications. Here, we report a case of mpox in a 52-year-old female with background diabetes mellitus and rheumatoid arthritis on 18 months of immunosuppressive drugs (prednisolone and methotrexate) who presented to an mpox treatment center at the University of Port Harcourt Teaching Hospital, Rivers State, Nigeria with disseminated febrile rash syndrome and a history of sexual exposure with a heterosexual partner with a febrile rash. Monkeypox virus (MPXV) and varicella-zoster virus (VZV) DNA levels in lesional swabs were quantified by qPCR, which returned positive and negative, respectively. Genomic sequencing was performed, and clade 2b was identified as the infecting clade of the virus. She was seronegative to HIV 1 and 2 (ELISA). Despite having a high burden of skin rash (>500 lesions), confluent skin distribution, and systemic complications, she essentially recovered on supportive treatment, highlighting her reduced net state of immunosuppression. This case report of mpox in a Nigerian lady with background immunosuppression successfully managed by a multidisciplinary team underscores the need for proactive screening and timely management of mpox to prevent unfavorable outcomes. Further studies are needed to understand the burden and outcome of mpox in the non-HIV immunosuppressive population.

## Introduction

Mpox (formerly monkeypox) is a zoonotic infection caused by the monkeypox virus (MPXV) of the Orthopoxvirus genus and the family Poxviridae [1]. There are 2 distinct clades of the virus: Clade II (previously known as the West African group) typically causes milder clinical disease and fewer fatalities than Clade I (previously known as the Central African (Congo Basin) group), which is associated with a more severe clinical course and higher mortality [1]. The exact animal(s) that serve as reservoir hosts for MPXV is not known, although rodents and nonhuman primates-such as rope squirrels, tree squirrels, Gambian pouched rats, dormice, and Macaca species of monkeys have all been implicated. Transmission of MPXV occurs through direct contact with infected animals, and from person-to-person contact through respiratory droplets, or inadvertent or intentional contact with contaminated fomites and surfaces [2]. The disease incubation period is usually 5-21 days, depending on the infecting clade of the virus, host immune status, and transmission route [2].

Mpox typically begins with systemic symptoms such as fever, headache, muscle pain, back pain, and fatigue, followed by multiple papules, blistering, pustules, ulcerative lesions on the face and body, and enlarged lymph nodes. Most patients with mpox recover within 3 to 4 weeks of the onset of skin rash, either spontaneously or requiring supportive treatment. However, severe illness with complications such as pneumonia, encephalitis, sepsis, and secondary bacterial infections can sometimes result in death among children, the elderly, pregnant women, and persons with chickenpox coinfection [3].

Individuals with compromised immune systems are at a higher risk of spreading infection and having severe disease and mortality because they cannot produce a protective immunological response, such as a neutralizing IgG and poxvirus-specific Th1 response [4]. The immune system plays a vital role in defending the body against microorganisms and other foreign bodies. The severity of the underlying disease, duration of illness, clinical severity, complications, comorbidities, or any potentially immune-suppressing treatment all play a role in determining the level of immunity in a patient [5]. Immunosuppression has been defined by Dohms and Saif as “a state of temporary or permanent dysfunction of the immune response resulting from insults to the immune system and leading to increased susceptibility to disease” [6]. Immunosuppression can be primary such as DiGeorge syndrome, Wiskott-Aldrich syndrome, HIV, or secondary such as haematologic malignancies, haematopoietic stem cell transplant, solid-organ transplant, severe autoimmune disorder, and medications such as high-dose corticosteroids (i.e. > or =20mg of prednisolone for two or more weeks), alkylating agents, antimetabolites, and biologic agents [4].

Although there have been reports of mpox cases in people living with HIV (PLHIV) [7], a paucity of cases exists for non-HIV immunosuppressed patients such as solid organ transplant recipients, haematologic malignancies, severe autoimmune disease, etc. Here, we report a rare case of mpox in a 53-year-old female with background diabetes mellitus and rheumatoid arthritis on immunosuppressive medications for the past 18 months who was successfully managed in an mpox treatment center in South-South Nigeria.

## Patient and Observation

**Patient information:** a 52-year-old female trader presented at the mpox treatment center of the University of Port Harcourt Teaching (UPTH), Port Harcourt, Nigeria, on 23 September 2024, with high-grade intermittent fever and chills for 7 days with associated headache, myalgia, back pain, and sore throat. About 4 days after the onset of febrile illness, she developed painful and itchy vesiculopustular skin eruptions that initially involved her forearms and then rapidly became generalized with the affectation of her trunk, groin, lower limbs, and vulvar (genitals) with oral lesions. She self-identified as being in a heterosexual relationship and had a condomless sexual exposure with a male partner who had a fever and oral lesions 5 days before the onset of symptoms. She also reported a history of urinary frequency and dysuria. There was no contact with animals or travel outside the state or country in the past 3 weeks. She reported a history of chickenpox infection in childhood. Her medical history reveals that she is a known hypertensive, living with type 2 diabetes mellitus (DM), and has been under management for rheumatoid arthritis (RA) diagnosed in February 2023. She is a widower and lives with her teen daughter in a 2-bedroom apartment.

**Clinical findings:** on admission, a physical examination reveals a very anxious woman with swollen axillary lymph nodes, hyperaemic lesions in the palate, and bilateral ankle oedema with an unremarkable systemic examination. Clinical signs comprised a temperature of 37.9°C, respiratory rate of 18 per minute, pulse rate of 93 per minute, and blood pressure of 110/60mmHg. Oxygen saturation (SP02) was 98% in room air.

**Diagnostic assessment:** her skin swab lesions were taken for real-time polymerase chain reaction (RT-PCR) of the monkeypox virus (MPXV) to the National Reference Laboratory (NRL) of the Nigerian Center for Disease Control and Prevention (NCDC). She was seronegative to HIV 1 and 2 (ELISA). Other laboratory parameters were presented (Table 1).

**Table 1:** laboratory parameters of the patient

S/No	Parameters	Result (on presentation)	Normal ranges	Interpretation	Result (after 4 days)	Interpretation
**1**	**Full blood count**					
	Packed cell volume (%)	31.30	37 – 47	Anaemia	34.20	Anaemia
	Platelet (106/L)	225	150 – 400	Normal	84	Thrombocytopenia
	Erythrocyte sedimentation rate (mm/hr)	112	5 – 7	Markedly Elevated	72	Markedly Elevated
	White blood cell (106/L)	5.1	4 – 10	Normal	7.4	Normal
	Neutrophil (%)	63.30	50 – 70	Normal	47.00	
	Lymphocyte (%)	29.90	20 – 40	Normal	44.50	Lymphocytosis
	Monocyte (%)	4.90	3 – 12	Normal	5	Normal
	Eosinophil (%)	1.80	0.5 – 5	Normal	2.70	Normal
	Basophil (%)	0.10	0 – 1.5	Normal	0.80	Normal
**2**	**Random blood glucose (mmol)**	4.43	3.5 - 5.0	Normal	4.83	Normal
**3**	**Serum electrolyte, urea and creatinine**					
	Sodium (mmol/1)	138	135 – 145	Normal		
	Potassium (mmol/1)	4.2	3.5 – 5.5	Normal		
	Bicarbonate mmol/1)	23	24 – 30	Reduced		
	Urea (mmo1/1	5	2.4 – 6.2	Normal		
	Creatinine (umo1/l)	105	60 – 130	Normal		
**4**	**HIV I&II screening (ELISA)**	Seronegative				
**5**	**Hepatitis B surface antigen**	Negative				
**6**	**Hepatitis C antibody**	Negative				

**Diagnosis:** based on the clinical and epidemiological information, a preliminary diagnosis of severe mpox in a non-HIV immunosuppressed host to exclude varicella-zoster virus (VZV) infection and secondary syphilis was made.

**Therapeutic interventions:** she initially refused admission due to fear of stigmatization. Three days later, she accepted hospital admission following counseling and worsening of her symptoms. The treatment regimen comprised of IV Ceftriaxone Ig 12 hourly, Tabs Amlodipine 10mg daily, Tabs Metformin 1g daily, Tabs Frusemide 40mg daily, Tabs Co-codamol 8/500mg 2 tabs twice a day, Tabs Pregabalin 75mg daily, Tabs Methotrexate 12.5mg weekly, Tabs Folic acid 10mg weekly, Tabs Prednisolone 5mg daily, Tabs Hydroxychloroquine 200mg twice a day (following a review by the rheumatologist), Tabs vitamin C 100mg thrice a day, Tabs Zinc 100mg daily. A dermatology review included cleaning skin lesions with normal saline and dilute povidone-iodine twice a day, daily chlorhexidine mouthwash, sitz bath twice a day, and topical mupirocin for open skin lesions.

**Follow-up and outcome of interventions:** on day 2 of hospitalization, she developed seizures and an acute confusional state and was reviewed by the Neuropsychiatry team, who placed her on Haloperidol IM 2.5mg 12 hourly for 3 days, Tabs Levetiracetam 500mg daily, and psychoeducation. On day 4 of hospitalization, her MPXV RT-PCR returned positive with a cycle threshold value of 15.84, and subsequent genomic sequencing of her skin specimen identified clade 2b variant, which is the circulating variant in Nigeria. However, RT-PCR for VZV was undetected. She was discharged after an 8-day hospital stay, following remarkably improved skin lesions and normal vital signs. She was seen one week after discharge at the mpox treatment center with resolving skin lesions, and was asked to continue follow-up at the infectious disease unit and rheumatology clinic.

**Patient´s perspective:** she was grateful for the treatment and care she received for her illness.

**Informed consent:** it was obtained for this publication.

## Discussion

As far as we know, this is the first reported case of mpox on background RA and immunosuppressive medications. Dohms and Saif in 1984 defined immunosuppression as “a state of temporary or permanent dysfunction of the immune response resulting from insults to the immune system and leading to increased susceptibility to disease” [6]. Immunosuppression can be primary such as DiGeorge syndrome, Wiskott-Aldrich syndrome, HIV, or secondary such as haematologic malignancies, haematopoietic stem cell transplant, solid-organ transplant, severe autoimmune disorder, and medications such as high-dose corticosteroids (i.e. > or =20mg of prednisolone for two or more weeks), alkylating agents, antimetabolites, and biologic agents [4]. The index case with a laboratory-confirmed mpox and a medical history of DM and RA on immunosuppressive medications (prednisolone and methotrexate) for the past 18 months with seronegative HIV is consistent with the diagnosis of mpox in a non-HIV immunosuppressed host. The demographic characteristics of the index case (female gender and above 50 years of age) differ from studies conducted during the 2017 and 2022 outbreaks in Nigeria which predominantly affected young adult men between 31 and 40 years while the identification of sexual exposure with a partner with “oral and genital lesion” aligns with epidemiologic studies that reported human-to-human transmission through sexual and non-sexual (household) contact as the major route of MPXV transmission in Nigeria [8].

The clinical presentation of an initial febrile prodrome accompanied a few days later by vesiculopustular skin eruptions and a localized genital lesion shows the mixed clinical picture of mpox, which may be due to different points of inoculation of MPXV (oral and genital lesions) during sexual activity [9]. Our index patient developed complications categorized based on body sites: skin complications (secondary bacterial skin infection), mucosal complications (pharyngitis, urethritis, and genital lesions), and systemic complications (encephalitis, neuropsychiatric complications). We found that neuropsychiatric complications such as encephalitis and anxiety observed in the index patient have been previously reported during the 2017 mpox reemergence in Nigeria by Ogoina et al. [10]. We relied on a constellation of symptoms (pharyngitis, fever, headache, adenopathy, vesiculopustular rash, and seizures) to make a clinical diagnosis of encephalitis, which was not confirmed by identifying MPXV in cerebrospinal fluid, an acknowledged limitation of this study. The psychiatric complications in mpox appear to follow events surrounding the disease process, including isolation and stigmatization. Healthcare workers must consider including adequate psychological counseling as an important component of mpox care. Additionally, the dissemination and amplification of accurate public health information are necessary for dispelling fear and misconceptions (infodemic) during infectious disease outbreaks such as mpox [11].

Most mpox studies in those with immunosuppression have been restricted to people living with HIV (PLHIV) and patients with uncontrolled viraemia who have been reported to have protracted illness, longer hospitalization, and a tendency to develop complications [7]. Individuals with compromised immune systems are at a higher risk of acquiring infections such as mpox and severe disease because they cannot produce a protective immunological response, such as a neutralizing IgG and poxvirus-specific Th1 response [4]. Severe outcomes, not always universal in immunosuppressed patients, have been attributed to the route of transmission, host immune response, type of clade, and the quantity of MPXV inoculated. Despite the high burden of skin rashes (>500 lesions), confluent skin distribution, systemic symptoms, and sole reliance on supportive treatment recorded in the index case, she essentially recovered within 30 days of the disease, reflecting her reduced net state of immunosuppression. This may be because RA is not considered a severe autoimmune disease and the fact that she was taking low doses of prednisolone and methotrexate under the recommendation of the rheumatologist emphasizing the diversity of the clinical presentation of mpox in immunocompromised hosts.

The role of a multidisciplinary team comprising infectious disease specialists, public health physicians, rheumatologists, dermatologists, mental health physicians, laboratory staff, and nurses underscores the place of multi-specialties in managing a complex mpox disease with complications and comorbidities. The fear of discrimination and stigmatization reported by our patient for which she initially refused hospital admission highlights the importance of patient education and health worker training in the management of epidemic-prone diseases such as mpox, especially in low-resource settings. Prevention of mpox is crucial in people with underlying immune suppression. However, it is unclear if mpox vaccination and other targeted public health interventions should be extended beyond PLHIV to non-HIV immunosuppressed hosts, since there is limited information on the impact of mpox in this population. There is a need to monitor the efficacy of the mpox vaccine (Modified vaccinia Ankara), particularly in immunocompromised patients where vaccine response may be suboptimal, and to further determine how many doses of the vaccine are required to generate sufficient IgG antibody response in this population.

## Conclusion

In summary, we report a rare case of laboratory-confirmed mpox in a Nigerian lady with background DM and RA who has been on immunosuppressive medications for the past 18 months and was successfully managed on supportive therapy. Proactive screening and treatment of mpox in patients with underlying immunosuppression are necessary to prevent unfavourable outcomes. Further studies are needed to understand the burden and impact of mpox in the non-HIV immunosuppressive population.
